# Association between Takayasu arteritis and ischemic heart disease: a cohort study

**DOI:** 10.31138/mjr.30.3.171

**Published:** 2019-09-30

**Authors:** Mathilde Versini, Shmuel Tiosano, Kassem Sharif, Naim Mahroum, Abdulla Watad, Doron Comaneshter, Guy Shalom, Yehuda Shoenfeld, Arnon D. Cohen, Howard Amital

**Affiliations:** 1 Department of Internal Medicine, Archet-1 Hospital, University of Nice-Sophia-Antipolis, Nice, France,; 2 Department of Medicine ‘B’, Sheba Medical Center, Tel Hashomer, Israel,; 3 Zabludowicz Center for Autoimmune Diseases, Sheba Medical Center, Tel Hashomer, Israel,; 4 Sackler Faculty of Medicine, Tel-Aviv University, Israel,; 5 Department of Quality Measurements and Research, Chief Physician’s Office, Clalit Health Services, Tel Aviv, Israel,; 6 Faculty of Health Sciences, Ben-Gurion University of the Negev, Beer-Sheva, Israel,; 7 Department of Dermatology and Venereology, Soroka Medical Center, Beer-Sheva, Israel,; 8 Siaal Research Center for Family Medicine and Primary Care, Faculty of Health Sciences, Ben-Gurion University of the Negev, Beer-Sheva, Israel

**Keywords:** Takayasu arteritis, ischemic heart disease, vasculitis, immune diseases

## Abstract

**Purpose of the study:**

Takayasu arteritis (TA) is an idiopathic large vessel vasculitis, which involves the aorta and its major branches. Our aim was to examine the association between TA and the development of ischemic heart disease (IHD) and its impact on survival.

**Study design:**

Using data from Clalit Health Services (CHS), the largest Health Maintenance Organization (HMO) in Israel, the proportion of IHD was compared between patients diagnosed with TA and age- and gender-matched controls. Chi-square and t-tests were used for univariate analysis, and a logistic regression model was employed for multivariate analysis. Survival analysis was performed using Kaplan-Meier plots and cox regression.

**Results:**

The study included 155 TA patients and 755 age- and gender-frequency matched controls. The proportion of IHD in TA patients was increased in comparison with controls (32.3% and 8.9%, p<0.001). In multivariate analysis, IHD was associated with TA (OR=6.576, 95% CI: 4.09–10.64) and male gender (OR=2.29, 95% CI: 1.43–4.26). Survival analysis over 15 years of follow-up showed a higher proportion of all-causes mortality in the TA group. In a multivariate analysis, TA (HR=2.58, 95%CI: 1.64–4.06) and IHD (HR=1.64, 95%CI: 1.05–2.55) were found to be associated with reduced survival.

**Conclusions:**

TA patients present an increased proportion of IHD, and a reduced 15-years survival rate compared to controls.

## INTRODUCTION

**What is already known:**

- Takayasu arteritis is a rare, chronic large-vessels vasculitis of unknown aetiology.
- It mainly affects women usually between ages 10–40.
- Coronary heart involvement is demonstrated in up to 60% of the patients either by angiography or by cardiac MRI

**The study’s main message**

- The proportion of IHD is increased among Takayasu patients, even after controlling for age and gender.
- Both Takayasu and IHD were found to be significantly, independently associated with long-term mortality after over 15 years of follow-up.

Takayasu arteritis (TA) is a rare, chronic large-vessels vasculitis of unknown aetiology characterized by granulomatous panarteritis of the aorta and its major branches. Histopathology reveals adventitial thickening, focal leukocytic infiltration of the tunica media and intimal hyperplasia, leading to the development of stenotic and aneurysmal lesions. Stenotic lesions are found in >90% of patients whereas aneurysms are reported in approximately 25%.^1^ It mainly affects women (80 to 90% of cases) usually between 10 and 40 years. A greatest prevalence of TA is observed in the Far East.^2^

Cardiac manifestations include myocarditis, aortic valvular regurgitation and coronary vessel stenosis which may lead to myocardial infarction, heart failure or sudden death.^3^

Coronary heart involvement is demonstrated in up to 60% of the patients either by angiography or by cardiac magnetic resonance imaging (MRI).^4–6^ However it becomes symptomatic in only 5–20% of the cases.^6^ Myocardial ischemic disease is considered as a major cause of morbidity and mortality in TA patients.^7,8^

The surgical management of coronary involvement is subject to frequent complications and relapses. There is therefore a major need to better understand this condition so as to allow early diagnosis and medical immunosuppressive treatment.

The aim of our study was to assess the proportion and prognostic significance of IHD among cohort of TA patients and matched controls, in a “real-life” population using Israel’s largest healthcare provider’s database.

## MATERIALS AND METHODS

The study was designed as a retrospective cohort study using data miming techniques from the Clalit Health Services (CHS) database. CHS is the largest managed care organization in Israel, serving a population of about 4,400,000 enrollees. CHS has a comprehensive computerized database that has a continuous real-time input from pharmaceutical, medical and administrative computerized operating systems.

Patients were defined as having Takayasu arteritis (TA), where there was at least one documented diagnosis of TA in the medical records registered by a physician practicing Internal Medicine, Rheumatology, Physical and Rehabilitation Medicine, Cardiology, Obstetrics and Gynaecology, Vascular Surgery, Ophthalmology and General Surgery, or when TA was listed in the diagnosis of discharge records following a hospitalization. Ischemic heart disease (IHD) was defined in a similar manner. Data available from CHS database included age, gender, BMI, smoking status, socioeconomic status (SES) and diagnosis of chronic diseases, including input date into CHS database. The validity of the diagnosis in the registry was found to be high in previous studies.^9–12^ Five control patients were randomly selected for each TA case. All patients were matched by age and gender. Matching was performed on date of registry in CHS database. The control group was selected from the list of CHS members, excluding patients with TA.

The age mentioned in the study relates to the entry age into the electronic records. However, we lacked the accurate age of disease onset. It should be remembered that in many patients the disease onset occurred many years antecedent to the electronic registry development. The Chi-square test was used to assess the distribution of categorical variables between TA patients and controls while the t-test was applied for continuous variables. A multivariate logistic regression model was used to explore the association between covariates including TA with IHD. Odds ratios (ORs) as well as confidence intervals (CIs) are presented. Survival analysis was performed using Kaplan-Meier plots and log-rank test. Follow-up time and person-years were calculated since TA diagnosis registration in CHS database until all-cause mortality, or January 1, 2017, whichever came first. Multivariate cox proportional hazards model was built to seek association between study covariates, including TA to IHD. Statistical analysis was performed using R Statistical Software (version 3.3.0; R Foundation for Statistical Computing, Vienna, Austria).

## RESULTS

The study included 155 TA patients and 775 age- and gender-frequency matched controls. Characteristics of the study population are presented in *Table 1*. The mean age in both groups was 47 years, with a predominance of women (87.7%). The proportion of IHD in patients with TA was increased compared with the proportion in controls (32.3% and 8.90%, p<0.001).

**Table 1.** Descriptive characteristics of the study population (n=930).

**Characteristics**	**Patients with TA (n=155)**	**Controls (n=775)**	**OR (95% CI)**	***p* value**
*Age (years) Mean ± SD	47.0 ± 18.8	47.0 ± 18.8	1.00 (0.99–1.01)	0.991
Female gender, n (%)	136 (87.7%)	680 (87.7%)	0.99 (0.60–1.73)	0.982
SES, n (%)				
*Low*	60 (39.2%)	301 (39.6%)	REF	REF
*Medium*	65 (42.5%)	310 (40.8%)	1.05 (0.71–1.55)	0.798
*High*	28 (18.3%)	149 (19.6%)	0.95 (0.57–1.53)	0.822
BMI Mean ± SD	25.9 ± 6.02	27.4 ± 11.5	0.97 (0.94–1.00)	0.047
IHD	50 (32.3%)	69 (8.90%)	4.89 (3.19–7.38)	<0.001

BMI: Body Mass Index; CI: Confidence interval; OR: Odds Ratio; SES: Socioeconomic status; TA: Takayasu Arteritis; IHD: Ischemic heart disease.

In *Table 2*, we report covariates associated with IHD. Both TA (OR=6.58, 95% CI: 4.09–10.64, p<0.001) and male gender (OR=2.29, 95% CI: 1.43–4.26, p<0.001) were found to be associated with IHD.

**Table 2.** Multivariate logistic regression: covariates associated with IHD.

	**OR**	**CI**	***p* value**
**Takayasu arteritis**	6.576	4.095–10.643	<0.001
**Age**	1.058	1.045–1.073	<0.001
**Gender (male)**	2.493	1.434–4.259	<0.001

CI: Confidence interval; OR: Odds Ratio.

**Figure 1.**
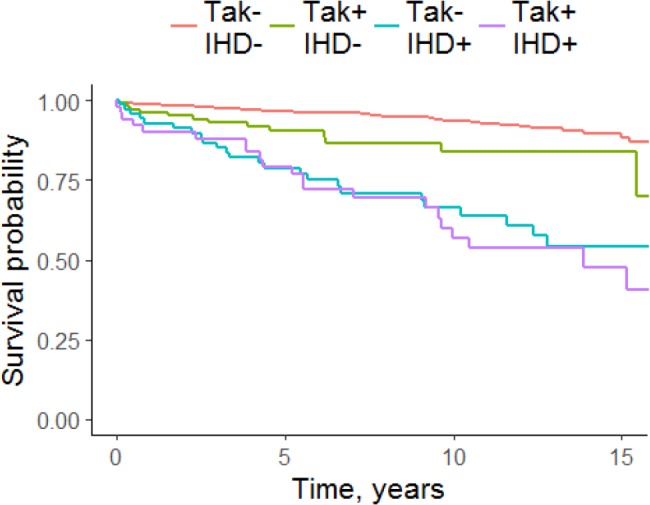
Kaplan-Meier survival analysis of Takayasu (Tak+) and control (Tak−) groups with (IHD+) and without (IHD−) ischemic heart disease.

*Figure 1* presents the Kaplan-Meier survival analysis over 15 years with a follow-up duration of 7585 person-years (log-rank test <0.01). A higher proportion of all-cause mortality was observed in the TA group when compared with the controls (21.9% and 8.77%, p<0.001). In multivariate cox regression analysis (*Table 3*), TA (HR=2.58, 95%CI: 1.64–4.06, p<0.001) and IHD (HR=1.64, 95%CI: 1.05–2.55, p=0.029) appeared to be associated with lower survival.

**Table 3.** Cox proportional hazards model for all-cause mortality.

	**HR**	**CI**	***p* value**
**Takayasu arteritis**	2.58	1.64–4.06	< 0.001
**IHD**	1.64	1.05–2.55	0.029
**Age**	1.07	1.06–1.09	< 0.001
**Gender: Male**	1.52	0.94–2.44	0.087

HR: Hazards ratio; CI: Confidence interval; IHD: Ischemic heart disease.

## DISCUSSION

In our study, ischemic heart disease was found to be independently associated with TA. Cardiac disease is a leading cause of morbidity and mortality in TA.^7,8^ Any anatomical structure of the heart may be affected, ranging from the pericardium to cardiac valves, myocardium and coronary arteries.^6^ Almost half of the patients have cardiac involvement at some point during the course of their disease.^6^ However, cardiac involvement is not clinically evident, since related symptoms are frequently absent in TA patients.^13^

The most common cardiac manifestation in TA is aortic regurgitation reported in 15% to 50% of patients, due to annular dilatation produced by ascending aortitis and aneurysm formation. Nevertheless, coronary involvement as first reported by Frovig and Loken in 1951,^14^ is not rare and has long been underestimated. In 1992, coronary artery involvement was thought to affect 9 to 10% of the TA patients 15]. Over the next two decades, it was estimated to exist in about 10–30% of the patients.^2^ In 2014, Kang et al.^5^ detected coronary arterial abnormalities in 53.2% of 111 TA patients by using coronary computed tomographic angiography. Henceforth we know that up to 60% patients are found to have coronary artery involvement.^5^ Despite the frequency of coronary artery lesions, it becomes symptomatic in only 5 to 20% of the cases. When present, manifestations include angina, acute myocardial infarction, arrhythmia, conduction abnormalities or congestive heart failure. In our study, 32.3% of TA patients presented with IHD compared with 8.9% of the controls. This is in agreement with the results reported in the literature. It is possible, however, that coronary involvement is still underestimated in our cohort, since, as mentioned previously, it is frequently asymptomatic.

In 1992, on the basis of clinical and pathological features, Matsubara et al.^15^ distinguished three types of coronary artery lesions: type 1 is stenosis or occlusion of the coronary ostia and the proximal segment of the coronary arteries; type 2 is diffuse or focal coronary arteritis which may extend diffusely to all epicardial branches or may involve focal segments; type 3 is coronary aneurysms.^15^ Type 1 is the commonest feature reported in >70% of TA patients^6^ while type 2 and type 3 coronary lesions are rare.^15^ However, several studies systematically investigating coronary artery disease suggest that type 2 lesions (middle and distal segments) are not so rare, non-ostial stenosis being present in 29–36% of cases, usually associated with ostial or proximal lesions.^5,16^

Vascular inflammation is the main mechanism of coronary arteriopathy. Narrowing of coronary ostia and proximal segments (type 1 lesions) is mainly due to extension of the adjacent inflammatory processes that occur in the ascending aorta (proliferation of the intima, contraction of the fibrotic media and adventitia). Premature atherosclerosis is also a major adjuvant factor. Seyahi et al.^17^ reported that more atherosclerotic plaques were observed in patients with TA (27%) than in healthy controls (2%). The fact that long-lasting vasculitis results in accelerated atherosclerosis is due to multiple factors such as chronic inflammation, secondary hypertension and long-lasting corticosteroid therapy.^17,18^ Additionally, men are recognized as being at a higher risk for atherosclerotic disease, especially IHD. We find this data in our study since IHD was significantly associated with male gender (OR=2.29).

Coronary arteriopathy is not the only mechanism of IHD. Myocardial scintigraphy and cardiac MRI, by using demonstrated myocardial ischemic disease without large coronary vessel abnormalities, highlight the possible involvement of coronary microcirculation.^4,13^ Myocardial perfusion defects on myocardial scintigraphy and myocardial scarring on cardiac MRI have been observed in 53% to 78% and 25% to 27%, respectively, of TA patients.^19–21^ Recently, Comarmond et al.^4,13^ investigated myocardial perfusion in 25 patients. They found a high prevalence (84%) of myocardial perfusion defects by scintigraphy mostly improved after vasodilation by dipyridamole. Coronary stenosis were present in only 18.2% of the patients, thus showing the reversibility of the perfusion defects by a vasodilatator agent and confirming the role of microcirculatory dysfunction in TA. Histological findings of myocardial biopsies in TA patients have demonstrated the presence of lymphocytic infiltration and myocyte necrosis.^6,13^

Considering the various mechanisms involved in IHD, it appears that both coronary and myocardial explorative imaging are necessary to evaluate cardiac disease in TA patients. Regarding coronary arteries, angiography remains the gold standard, but coronary CT angiography can be an interesting non-invasive option. Additionally, myocardial function should be evaluated by using scintigraphy or cardiac MRI.

Cardiac disease is the most important cause of mortality in TA patients.^7,8^ Deaths are due to congestive heart failure, acute myocardial infarction or sudden death.^2^ Park et al.,^8^ in a large population-based study, recently reported cardiovascular disease as the leading cause of mortality accounting for 45% of the deaths in TA patients.^8^ The overall survival of TA patients was found to be significantly lower than the general population; our study clearly corroborates this observation.

Consequently, it is ideally necessary to establish the diagnosis at the early phase of the disease, especially in the pre-stenotic phase and to introduce immunosuppressive therapy. Despite the paucity of evidence due to the lack of controlled trials, corticosteroids often combined with other immunosuppressive steroid-sparing agent are indicated when active vasculitis is suspected.^1^ When revascularization is necessary, several options can be considered and requires the expertise of an experienced cardiovascular surgeon. Coronary artery bypass grafting (CABG), balloon angioplasty followed by stenting, and transaortic endarterectomy are used depending on the characteristics of the lesions.^6,22^ Surgical treatment should be delayed, if possible, during the active inflammation period, until clinical remission is achieved. The incidence of 5- and 10-years restenosis is significantly higher after angioplasty (10–80%) than after CABG (5–40%).^23,24^ Myocarditis without evidence of coronaropathy requires immunosuppressive therapy combined with conventional heart failure therapy. Our study has several strengths. First, TA is a rare disease, so large cohorts are rare; our TA cohort is one of the largest. In addition, CHS database reflects “real-life” patients, breaking out the stringent inclusion criteria of studies. Furthermore, in contrast to a few authors that investigated cardiac involvement in TA, we have an age- and gender-matched control group that permits proper statistical analysis. Our study has several limitations. This database was designed for clinical use, and therefore we cannot assure that the diagnoses strictly fulfil the diagnostic criteria of TA and IHD. However, the validity of diagnosis of this type of study was demonstrated in previous studies.^9–12^ Moreover, our results are comparable to those observed in the literature, confirming the quality of our data. The design of our study does not allow us to conclude cause-and-effect relationship between IHD and TA.

In conclusion, TA was found to be a significantly associated with IHD. Furthermore, TA patients appear in our cohort to have a lower 15-years survival rate.

## Data Availability

**Ethics committee approval:** Ethical approval was obtained from the CHS institutional ethics review board. The study was exempted from singing informed consent forms.

## ABBREVIATIONS

CABG: Coronary Artery Bypass Grafting
CHS: Clalit Health Services
CIs: Confidence Intervals
IHD: Ischemic Heart Disease
MRI: Magnetic Resonance Imaging
ORs: Odds Ratios
SES: Socioeconomic Status
TA: Takayasu Arteritis
